# *Elizabethkingia anophelis* bacteremia is associated with clinically significant infections and high mortality

**DOI:** 10.1038/srep26045

**Published:** 2016-05-17

**Authors:** Susanna K. P. Lau, Wang-Ngai Chow, Chuen-Hing Foo, Shirly O. T. Curreem, George Chi-Shing Lo, Jade L. L. Teng, Jonathan H. K. Chen, Ricky H. Y. Ng, Alan K. L. Wu, Ingrid Y. Y. Cheung, Sandy K. Y. Chau, David C. Lung, Rodney A. Lee, Cindy W. S. Tse, Kitty S. C. Fung, Tak-Lun Que, Patrick C. Y. Woo

**Affiliations:** 1 Department of Microbiology, The University of Hong Kong, Hong Kong, China; 2 State Key Laboratory of Emerging Infectious Diseases, The University of Hong Kong, Hong Kong, China; 3 Research Centre of Infection and Immunology, The University of Hong Kong, Hong Kong, China; 4 Carol Yu Centre for Infection, The University of Hong Kong, Hong Kong, China; 5 Department of Pathology, United Christian Hospital, Hong Kong, China; 6 Department of Pathology, Pamela Youde Nethersole Eastern Hospital, Hong Kong, China; 7 Department of Pathology, Kwong Wah Hospital, Hong Kong, China; 8 Department of Clinical Pathology, Tuen Mun Hospital, Hong Kong, China

## Abstract

Unlike *Elizabethkingia meningoseptica*, the clinical importance of *E. anophelis* is poorly understood. We determined the clinical and molecular epidemiology of bacteremia caused by *Elizabethkingia-*like species from five regional hospitals in Hong Kong. Among 45 episodes of *Elizabethkingia*-like bacteremia, 21 were caused by *Elizabethkingia*, including 17 *E. anophelis*, three *E. meningoseptica* and one *E. miricola*; while 24 were caused by other diverse genera/species, as determined by 16S rRNA gene sequencing. Of the 17 cases of *E. anophelis* bacteremia, 15 (88%) were clinically significant. The most common diagnosis was pneumonia (n = 5), followed by catheter-related bacteremia (n = 4), neonatal meningitis (n = 3), nosocomial bacteremia (n = 2) and neutropenic fever (n = 1). *E. anophelis* bacteremia was commonly associated with complications and carried 23.5% mortality. In contrast, of the 24 episodes of bacteremia due to non-*Elizabethkingia* species, 16 (67%) were clinically insignificant. Compared to non-*Elizabethkingia* bacteremia, *Elizabethkingia* bacteremia was associated with more clinically significant infections (*P* < 0.01) and positive cultures from other sites (*P* < 0.01), less polymicrobial bacteremia (*P* < 0.01), and higher complication (*P* < 0.05) and mortality (*P* < 0.05) rates. *Elizabethkingia* bacteremia is predominantly caused by *E. anophelis* instead of *E. meningoseptica*. *Elizabethkingia* bacteremia, especially due to *E. anophelis*, carries significant morbidity and mortality, and should be considered clinically significant unless proven otherwise.

The genus *Elizabethkingia* comprises aerobic, non-fermenting, non-motile and non-spore-forming gram-negative rods that were previously named *Flavobacterium* or belonged to CDC group IIa and later reclassified as *Chryseobacterium* in 1994^1^. In 2005, *Chryseobacterium meningosepticum* and *C. miricola* were transferred to a new genus, *Elizabethkingia*, on the basis of combined phenotypic and phylogenetic characteristics^2^. The genus comprises three medically important species, *Elizabethkingia anophelis*, *E. meningoseptica* and *E. miricola*. A novel species, *E. endophytica*, isolated from sweet corn, was also recently proposed^3^. *E. meningoseptica,* previously named *Flavobacterium meningosepticum* or *C. meningosepticum*, is the best known species among the genus. *E. meningoseptica* is a causative agent of nosocomial infections especially in immunocompromised patients, as well as neonatal meningitis and sepsis^4^. Besides soil, fresh water and plants, the bacterium can be found in hospital environments and may contaminate flushing solutions and medical devices^5^. Infections caused by *E. meningoseptica* can be difficult to treat and carry high mortalities, which may be partly explained by their intrinsic multidrug resistance towards commonly used antibiotics such as β-lactams and aminoglycosides^6^. Therefore, accurate diagnosis is important to guide appropriate antibiotic regimens which often consist of a combination of ciprofloxacin or rifampicin with piperacillin-tazobactam or vancomycin.

In contrast to *E. meningoseptica*, the epidemiology and pathogenicity of *E. anophelis* and *E. miricola* were less well understood. *E. miricola*, originally named *C. miricola* when first isolated from condensed water obtained from the Russian space station, Mir, only rarely causes nosocomial infections in human^7^,^8^. On the other hand, *E. anophelis* was first isolated from midgut of the mosquito *Anopheles gambiae* in 2011^9^. Soon after its discovery, it was reported to cause neonatal meningitis in the Central African Republic and a nosocomial outbreak in an intensive-care unit in Singapore^10^,^11^. The first discovery of *E*. *anophelis* from mosquito gut has raised suspicion on mosquitoes as the source of neonatal meningitis cases in Africa^10^. However, our recent report on *E. anophelis* meningitis in two neonates and chorioamnionitis in a neonate’s mother in Hong Kong suggested that mosquitoes were unlikely the vehicles of transmission^12^. Since the transmission route was initially obscure, draft genome sequencing was performed and showed evidence for perinatal vertical transmission from a mother to her neonate^12^. The ultimate resolution power of genome sequencing also enabled species confirmation and discrimination from the phenotypically similar species, *E. meningoseptica*^12^.

Since *E. anophelis* was commonly misidentified as *E. meningoseptica* in previous reports^10^,^11^,^12^,^13^, we hypothesize that many previously described *E. meningoseptica* isolates were actually *E. anophelis* and that *E. anophelis* may account for a significant proportion of *Elizabethkingia* infections. To better understand the epidemiology and clinical disease spectrum of *E. anophelis* and *Elizabethkingia* as a whole, we determined the clinical and molecular epidemiology of bacteremia caused by *Elizabethkingia-*like species from five regional hospitals in Hong Kong. All bacteremia episodes caused by *Elizabethkingia-*like species identified by conventional phenotypic tests from 2004 to 2013 during the study period were included. For the 45 episodes of *Elizabethkingia*-like bacteremia identified, 16S rRNA gene sequencing was performed for species identification and clinical characteristics and outcomes were analyzed.

## Results

### Identification of *Elizabethkingia*-like bacteremia by 16S rRNA gene sequencing

**Figure 1:**
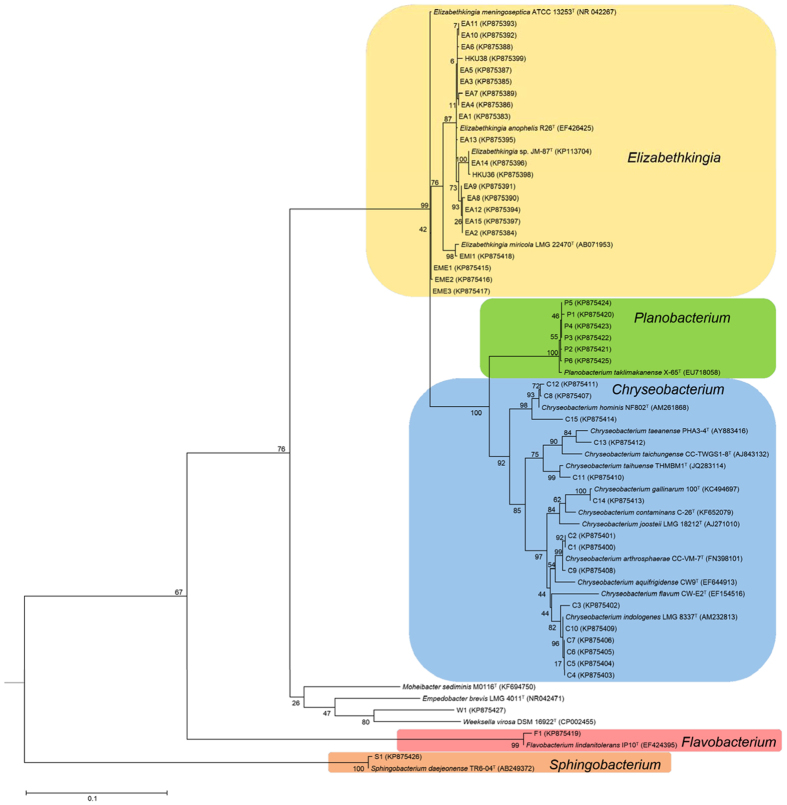
Phylogenetic tree showing the relationship of the 45 *Elizabethkingia*-like blood culture isolates to related bacterial species using 16S rRNA gene sequence analysis. The tree was constructed by maximum likelihood method using General Time Reversible model and *Escherichia coli* (CP010304) as the root. A total of 1325 nucleotide positions were included in the analysis. Bootstrap values were calculated from 1000 replicates. The scale bar indicates the number of substitutions per site. Names and accession numbers are given as cited in GenBank database.

Twenty-one of the 45 episodes of *Elizabethkingia*-like bacteremia were caused by *Elizabethkingia*, while 24 episodes were caused by diverse genera/species including *Chryseobacterium* (n = 15), *Flavobacterium* (n = 1), *Planobacterium* (n = 6), *Sphingobacterium* (n = 1) and *Weeksella*-like species (n = 1) according to 16S rRNA gene analysis (Fig. 1 and see Supplementary Table 1). Of the 21 episodes of *Elizabethkingia* bacteremia, 17 were caused by *E. anophelis* (99.0–99.9% nucleotide identity to *E. anophelis* type strain R26^T^), three by *E. meningoseptica* (99.4–99.8% nucleotide identity to *E. meningoseptica* type strain ATCC 13253^T^) and one by *E. miricola* (99.5% nucleotide identity to *E. miricola* type strain LMG 22470^T^).

Among the 24 episodes of non-*Elizabethkingia* bacteremia, 15 were caused by *Chryseobacterium* species, among which two (C13 and C15) represented two potentially novel *Chryseobacterium* species (≤98.4% nucleotide identity to existing *Chryseobacterium* species). One episode was caused by another potentially novel species most closely related to *Weeksella* (91.9% nucleotide identity to *Weeksella virosa* DSM 16922^T^). The other eight episodes were caused by *Planobacterium* (n = 6), *Flavobacterium* (n = 1) and *Sphingobacterium* (n = 1) species respectively.

### Clinical characteristics of patients with *Elizabethkingia*-like bacteremia

The clinical characteristics of the 45 episodes of *Elizabethkingia*-like bacteremia were summarized in Tables 1, 2, 3. Of the 17 episodes of *E. anophelis* bacteremia, the male:female ratio was 11:6, with a median age of 58 years (range, 1 day to 104 years) (Table 2). Most patients had underlying diseases. Except two patients with pseudobacteremia, most cases were associated with clinically significant bacteremia, with 12 cases being hospital-acquired and three community-acquired. The most common diagnosis was pneumonia (n = 5), followed by catheter-related bacteremia (n = 4), neonatal meningitis (n = 3), nosocomial bacteremia (n = 2) and neutropenic fever (n = 1). Among the five cases of pneumonia, three were community-acquired, including one in a 51-year-old previously healthy man (case EA10). All three cases of neonatal meningitis were hospital-acquired. Details of two (HKU36 and HKU38) of the three neonatal meningitis cases have been reported previously^12^. They were included because they fell into the inclusion criteria during the study period. The source of *E. anophelis* in the two cases of nosocomial bacteremia and one case of neutropenic fever was obscure, although severe mucositis was present in the latter. Three of the 17 patients had multiple positive blood cultures. *E. anophelis* was concomitantly isolated from other body sites in seven patients, including cerebrospinal fluid (n = 3), respiratory samples (n = 3) and catheter tip (n = 1). Most patients survived, but four patients died despite appropriate antibiotics given in two of the four patients. Complications as a result of *E. anophelis* bacteremia included acute pulmonary edema, congestive heart failure, multi-organ failure, disseminated intravascular coagulation, septic shock, haematemesis, acute renal failure, metabolic acidosis and intraventricular haemorrhage. Various antibiotic regimens as single drug or combinations were used for empirical or definitive treatment, including quinolones, penicillins, cephalosporins, carbapenems, vancomycin and rifampicin. Removal of catheter was often required in catheter-related bacteremia cases in addition to antibiotic treatment.

All the four patients with *E. meningoseptica* or *E. miricola* bacteremia also had underlying disease and clinically significant infections (Table 2). Two patients with *E. meningoseptica* bacteremia had biliary tract infections, while one had nosocomial bacteremia. Interestingly, the only episode of *E. miricola* bacteremia (case EMI1) was detected in the same patient with *E. anophelis* nosocomial pneumonia (case EA1) which occurred more than one month previously during the same admission and was complicated by heart failure and acute pulmonary edema requiring intubation and inotropes. Five weeks after successful treatment with piperacillin-tazobactam, the patient developed another episode of bacteremia caused by *E. miricola* which was also isolated from central venous catheter. The patient recovered with levofloxacin and catheter removal.

In contrast, 16 of the 24 patients with bacteremia due to non-*Elizabethkingia* species had pseudobacteremia (Table 3). The other eight patients were diagnosed to have nosocomial bacteremia (three cases), transfusion-related bacteremia (two cases), catheter-related bacteremia (one case), neutropenic fever (one case) and sepsis (one case). Except for a case of community-acquired sepsis in a 90-year-old man (case P1), all other cases were hospital-acquired. Only one episode of catheter-related bacteremia was complicated by septic shock, where *C. arthrosphaerae* was isolated from six blood cultures. All the eight patients survived.

Comparison between *Elizabethkingia* and non-*Elizabethkingia* cases showed that clinically significant infections were more common in patients with *Elizabethkingia* bacteremia than those with non-*Elizabethkingia* bacteremia (i.e. fewer pseudobacteremia cases were observed) (*P* < 0.01). Moreover, patients with *Elizabethkingia* bacteremia had more positive cultures from other sites (*P* < 0.05) and lower incidence of polymicrobial bacteremia (*P* < 0.01). They also showed higher complication (*P* < 0.05) and mortality (*P* < 0.05) rates than those with with non-*Elizabethkingia* bacteremia (Table 1).

### Microbiological characteristics of *Elizabethkingia* isolates

All the 21 *Elizabethkingia* isolates were non-motile, oxidase-positive, catalase-positive, indole-positive, non-glucose-fermenting, Gram-negative bacilli. Growth on MacConkey agar, citrate utilization, urea hydrolysis and fermentation of cellobiose and melibiose fermentation were variable. All 21 isolates were identified as *E. meningoseptica* by Vitek 2 GNI system with 91–99% confidence. All 21 isolates were susceptible to ciprofloxacin, cefoperazone-sulbactam and vancomycin, but were resistant or intermediate-resistant to imipenem, amikacin, gentamicin and tobramycin. Susceptibilities to ceftazidime, piperacillin, rifampicin and cotrimoxazole were variable (see Supplementary Table 2).

**Figure 2:**
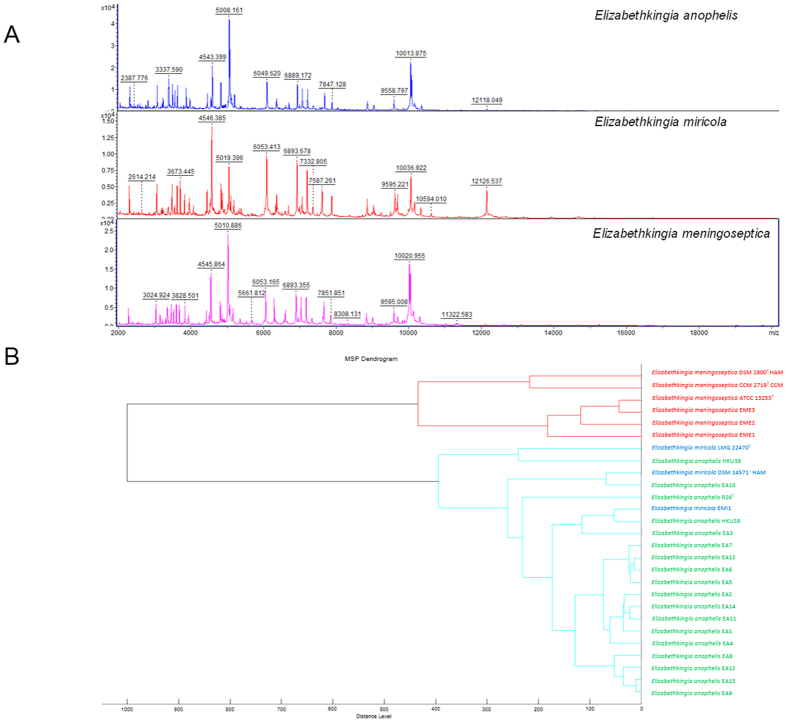
Results of MALDI-TOF MS identification of the 21 *Elizabethkingia* strains. In panel (**A**), representative MALDI-TOF MS spectra of the three *Elizabethkingia* species are shown. In panel (**B**), dendrogram was generated from hierarchical clustering of MALDI-TOF MS spectra of 21 *Elizabethkingia* isolates and reference strains of *E. meningoseptica* and *E. miricola*, using ClinProTools 3.0 (Bruker Daltonics, Germany). Distances are displayed in relative units.

MALDI-TOF MS using Reference Library Biotyper v3.1.2.0 (Bruker Daltonik, Germany) failed to identify the 17 *E. anophelis* isolates (10 misidentified as *E. meningoseptica* with score 2.073 to 2.403, two identified as *Elizabethkingia* species with score 1.952–1.971 and five unidentified with score 1.32 to 1.42 to *E. meningoseptica*). When the database was expanded with inclusion of mass spectra from seven *E*. *anophelis* isolates, all the other 10 *E. anophelis* isolates were correctly identified as *E. anophelis* with score 2.321 to 2.634. The three *E. meningoseptica* and one *E. miricola* strains were correctly identified by the Bruker reference library (see Supplementary Table 2). Hierarchical cluster analysis showed that the protein mass spectra of *E. anophelis* and *E. miricola* were clustered together but formed a distinct branch from *E. meningoseptica* (Fig. 2).

### PFGE typing of *E. anophelis*

**Figure 3:**
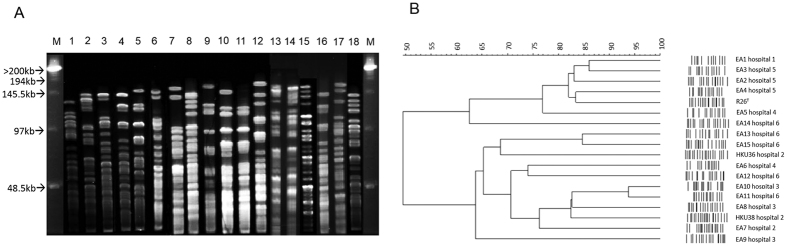
Pulsed-field gel electrophoresis (PFGE) analysis of the 17 *E. anophelis* isolates and *E. anophelis* type strain R26^T^. (lane 1 = EA1, lane 2 = EA2, lane 3 = EA3, lane 4 = EA4, lane 5 = EA5, lane 6 = EA6, lane 7 = EA7, lane 8 = EA8, lane 9 = EA9, lane 10 = EA10, lane 11 = EA11, lane 12 = EA12, lane 13 = EA13, lane 14 = EA14, lane 15 = EA15, lane 16 = HKU38, lane 17 = HKU36, lane 18 = R26^T^, M = lambda marker). In Panel (**A**), PFGE was performed using CHEF Mapper XA system (Bio-Rad) and restriction endonuclease *Xba*I. Results showed that the 17 isolates possessed distinct PFGE patterns. In Panel (**B**), dendrogram was constructed with PFGE data by similarity and clustering analysis using the Dice coefficient (1% tolerance and 0.5% optimization) and unweighted pair-group method using average linkages with GelCompar II.

To determine the genetic relatedness of the 17 *E. anophelis* strains isolated from five different hospitals, PFGE was performed using the bacterial genomic and DNA restriction endonuclease *Xba*I. In general, the PFGE patterns among the 17 *E. anophelis* strains were distinct from each other and different from that of the type strain R26^T^. Dendrogram constructed using the PFGE images showed that some adjacently clustered strains were isolated from the same hospital (Fig. 3). For example, strains EA2, EA3 and EA4 were from hospital 5, while strains EA13 and EA15 were from hospital 6. However, no clear epidemiological linkage could be identified in terms of time and place of blood culture isolation. Specifically, strains EA2, EA3 and EA4 were isolated from different years. Strains EA13 and EA15 were isolated four months apart and the two patients were hospitalized in different wards of hospital 6.

## Discussion

*E. anophelis* bacteremia should be considered clinically significant unless proven otherwise and should prompt appropriate antibiotic therapy. In contrast to the traditional belief that *E. meningoseptica* was the most important *Elizabethkingia* species associated with bacteremia, the present study showed that the majority of *Elizabethkingia* bacteremia cases were caused by *E. anophelis*. In particular, neonatal meningitis was associated exclusively with *E. anophelis* in this study. In the present series, *E. anophelis* bacteremia mainly occurred in neonates or adults with underlying medical illnesses, and was most commonly associated with pneumonia and hospital-acquired infections such as catheter-related bacteremia and neonatal meningitis. Notably, community-acquired pneumonia occurred in three patients including a healthy middle-aged adult, where the infective source remained obscure. Apart from the two previous reported cases of neonatal meningitis (HKU36 and HKU38) which were most likely acquired from maternal-to-infant transmission based on comparative genomics^12^, no obvious epidemiological linkage or genetic relatedness was identified among the other cases. Nevertheless, some potentially genetically related strains upon PFGE analysis may have circulated in the same hospital. Further studies should be performed to better understand the disease spectrum and transmission routes of *E. anophelis*.

*E. anophelis* bacteremia carries significant morbidity and mortality. Various complications were observed and four patients died, giving a mortality rate of 23.5%. This is in line with previous reports of *E. anophelis* neonatal meningitis being associated with poor outcomes^10^. Nevertheless, all the three present cases of neonatal meningitis were cured with early use of appropriate antibiotics^12^. Although *E. anophelis* usually confers resistance to multiple antibiotics such as ceftazidime, imipenem and aminoglycosides^12^, all the 17 isolates were susceptible to ciprofloxacin, cefoperazone-sulbactam and vancomycin, which should be considered in empirical treatment while awaiting susceptibility results. In cases of catheter-related bacteremia, infected catheters should be removed in addition to antibiotic treatment. Future prospective studies with population-based data should be performed to determine the prevalence or incidence of *E. anophelis* bacteremia.

*E. meningoseptica* and *E. miricola* appeared to be much less prevalent than *E. anophelis*, although similar studies in other countries are required to more accurately assess their relative importance. Similar to *E. anophelis*, *E. meningoseptica* and *E. miricola* bacteremia were associated with clinically significant infections. Besides one case of *E. meningoseptica* nosocomial bacteremia and one case of *E. miricola* catheter-related bacteremia, biliary tract infections were also noted in two cases of *E. meningoseptica* bacteremia. It remains to be determined if *E. meningoseptica* may have the propensity to cause biliary tract infections among the genus. Given their similar antibiotic susceptibility profiles to that of *E. anophelis*, ciprofloxacin, cefoperazone-sulbactam and vancomycin should also be included in treatment regimens for *E. meningoseptica* and *E. miricola* bacteremia.

In contrast to *Elizabethkingia* species, isolation of non-*Elizabethkingia* species from blood cultures should raise suspicion of their clinical significance. In this study, non-*Elizabethkingia* bacteremia is associated with higher incidence of pseudobacteremia and polymicrobial bacteremia than *Elizabethkiniga* bacteremia. In particular, all six isolates of *C. indologenes* and the three potentially novel species were associated with pseudobacteremia. Bacteremia caused by non-*Elizabethkingia* species is also associated with lower incidence of complications and mortality than *Elizabethkingia* bacteremia, suggesting that these bacterial species may be less virulent than *Elizabethkingia*. Moreover, these environmental, non-*Elizabethkingia* bacterial species may contaminate blood cultures when aseptic techniques during blood taking are breached. Careful clinical assessment is required to determine the clinical significance and the need for antibiotics when these bacteria are isolated from blood cultures.

Although the two *E. anophelis* strains, HKU36 and EA14, are phylogenetically genetically close to *E. endophytica* strain JM-87^T^, they should belong to *E. anophelis* instead of *E. endophytica* or a novel species. The two strains possessed 99.9% nucleotide identities to both the newly proposed species, *E. endophytica* strain JM-87^T^ and *E. anophelis* R26^T^ in their 16S rRNA gene sequences. Although strain JM-87^T^ was reported to possess 51–52% similarities to *E. anophelis* R26^T^ by DNA-DNA hybridization^3^, our previous study showed that the draft genome of strain HKU36 possessed 78.3% nucleotide identity to the genome of *E. anophelis* R26^T^ by estimation of intergenomic distance^12^. Given the close relatedness of strains HKU36, EA14 and JM-87^T^ in their 16S rRNA genes (Fig. 1), it is likely that the genome sequences of strain JM-87^T^ and EA14 may also possess >70% identity to that of *E. anophelis* R26^T^. Since genome-based comparison can offer ultimate resolution for species delineation which is superior to traditional DNA-DNA hybridization methods, genome sequencing of strain JM-87^T^ and related strains such as EA14 should be performed to more accurately define their taxonomic positions.

The present results confirmed our suspicion that *E. anophelis* was a previously under-reported bacterium which can be easily misidentified as *E. meningoseptica*^12^. Although *E*. *anophelis* was first discovered from mosquito gut^10^, we previously showed that maternal chorioamnionitis, instead of mosquitoes, was more likely the source of neonatal meningitis^12^. Similarly, mosquitoes are unlikely the route of transmission in other *E. anophelis* infections. We speculate that contaminated environments, such as infected catheters, are the source of infection in most cases, as in the case of previously described “*E. meningoseptica*” infections. Given their similar phenotypic characteristics, *E. anophelis* isolates from previous reports were often mistaken as *E. meningoseptica* initially^10^,^11^,^12^,^14^. Phenotypic tests, such as acid production from cellobiose and citrate utilization, previously reported as potentially useful for species discrimination^12^, were unlikely to be reliable. In our previous study, the three *E. anophelis* strains were also initially misidentified as *E. meningoseptica* even with MALDI-TOF MS, owing to the absence of *E. anophelis* spectra in commercial databases^12^. The 16S rRNA genes of *E. anophelis* possessed >98% nucleotide identity to those of *E. meningoseptica* and *E. miricola*, which should offer sufficient discriminative power^12^. However, some “*E. meningoseptica”* strains with 16S rRNA sequences deposited in GenBank, such as strains G3-1-08 and 502^15^,^16^, should belong to *E. anophelis* based on phylogenetic analysis^12^. These “misidentified” strains in GenBank may confuse the interpretation of 16S rRNA gene sequencing results and should be rectified^12^. While *E. anophelis* can be distinguished from *E. meningoseptica* by MALDI-TOF MS when the database is expanded with mass spectra from *E. anophelis* strains, *E. anophelis* and *E. miricola* appear to be indistinguishable from each other. With an expanded database using *E. anophelis* isolates, MALDI-TOF MS is the method of choice for rapid and accurate diagnosis of *E. anophelis* infections, which is crucial to better understand its epidemiology and clinical disease spectrum.

## Methods

### Ethics statement

The use of blood culture isolates and anonymous clinical data were approved by Institute Review Board, The University of Hong Kong/Hospital Authority, Hong Kong (reference UW 04-278 T/600). The methods and all experimental protocols were carried out in accordance with the approved guidelines. Since this study does not involve experimentation on human subjects or the use of tissue samples from human subjects, written informed consent has been waived by our institutional review board.

### Settings and Patients

Patients were hospitalized in five regional hospitals located in different areas of Hong Kong from 2004 to 2013. To identify potential cases of *Elizabethkingia* bacteremia, all bacteremia episodes caused by oxidase-positive, non-glucose fermenters that were identified as *Elizabethkingia*, *Flavobacterium* or *Chryseobacterium* species by conventional phenotypic tests during the study period were included with clinical data analyzed^17^. Bacteremia was categorized as clinically significant or pseudobacteremia (contamination of blood culture) by clinical and laboratory criteria^18^. The criteria include the patient’s clinical presentation, physical examination findings, body temperature at the time of the blood culture, leukocyte and differential cell counts, imaging or operative results, histopathological findings, number of positive blood cultures out of the total number performed, and response to treatment.

### Bacterial isolates

Collection of clinical specimens, bacterial cultures and conventional phenotypic identification were performed according to standard protocols^19^. Two of the 45 *Elizabethkingia*-like isolates from two neonates (HKU36 and HKU38) have been reported previously^12^. The same isolate recovered from the same patient was counted only once.

### 16S rRNA gene sequencing for species identification

The 45 blood culture isolates were subject to 16S rRNA gene sequencing according to previously published protocols with modifications, using primers LPW57 (5′-AGTTTGATCCTGGCTCAG-3′) and LPW58 (5′-AGGCCCGGGAACGTATTCAC-3′)^12^,^20^. The sequences of PCR products were compared to known gene sequences in GenBank by multiple sequence alignment using CLUSTAL_W in MEGA version 6^21^. Phylogenetic tree was constructed by maximum likelihood method using MEGA version 6^21^.

### Statistical analysis

A comparison of characteristics was made between patients with *Elizabethkingia* and non-*Elizabethkingia* bacteremia using Chi-square test (IBM SPSS Statistics version 19). *P* < 0.05 was regarded as statistically significant.

### Phenotypic characterization and matrix-assisted laser-desorption ionization-time-of-flight mass-spectrometry (MALDI-TOF MS) of *Elizabethkingia* isolates

The 21 *Elizabethkinigia* isolates were characterized by phenotypic tests, Vitek 2 GNI system (bioMérieux, France) and MALDI-TOF MS. MALDI-TOF MS was performed by ethanol formic acid extraction method as described previously, using Bruker Daltonics microflex LT system with Reference Library Biotyper v3.1.2.0 (Bruker Daltonik, Germany)^22^,^23^. Since *E. anophelis* is not included in the Bruker reference, mass spectra generated from seven *E. anophelis* strains with identity confirmed by 16S rRNA gene sequencing were later added to the database. Obtained spectra were subject to hierarchical cluster analysis as described previously^24^. Antibiotic susceptibility testing was performed by Kirby Bauer disk diffusion method with results interpreted according to Clinical and Laboratory Standards Institute for *Staphylococcus aureus* (vancomycin) and *Pseudomonas aeruginosa* (other drugs), because of the lack of interpretative criteria for *Elizabethkingia*^25^.

### Pulsed-field gel electrophoresis (PFGE)

The 17 *E. anophelis* isolates were characterized by PFGE using CHEF Mapper XA system (Bio-Rad, CA, USA) and restriction endonuclease *Xba*I as described previously^12^,^26^. After PFGE, the gel was stained with ethidium bromide (1 μg/ml) for 30 minutes and the patterns of the genomic DNA digest were visualized with a UV transilluminator. Digital images were stored electronically as TIFF files and analyzed visually and with GelCompar II (version 3.0; Applied Maths, Kortrijk, Belgium), and represented by UPGMA method.

### Nucleotide sequence accession number

The 16S rRNA gene sequences of the 45 blood culture isolates have been deposited in the GenBank sequence database under accession no. KP875383 to KP875427.

## Additional Information

**How to cite this article**: Lau, S. K. P. *et al.* *Elizabethkingia anophelis* bacteremia is associated with clinically significant infections and high mortality. *Sci. Rep.* **6**, 26045; doi: 10.1038/srep26045 (2016).

## Supplementary Material

Supplementary Tables

## Figures and Tables

**Table 1:** Characteristics of the 45 patients with bacteremia caused *Elizabethkingia-*like organisms

Characteristics	Number of patients (%)		*P*-value^c^
	*Elizabethkingia*bacteremia (n = 21)	Non-*Elizabethkingia*bacteremia (n = 24)	
Sex (male:female)	11:10	13:11	0.90
Underlying diseases	19 (90.5)	24 (100)	0.12
Hospital- vs community-acquired^a^	15:4	7:1	0.60
Diagnosis^b^			
Biliary tract infection	2 (9.5)	0 (0)	0.12
Catheter-related bacteremia	5 (23.8)	1 (4.2)	0.05
Community-acquired pneumonia	3 (14.3)	0 (0)	0.06
Neonatal meningitis	3 (14.3)	0 (0)	0.06
Neutropenic fever	1 (4.8)	2 (8.3)	0.63
Nosocomial bacteremia	3 (14.3)	5 (20.8)	0.57
Nosocomial pneumonia	2 (9.5)	0 (0)	0.12
Primary bacteremia	0 (0)	1 (4.2)	0.34
Pseudobacteremia	2 (9.5)	16 (76.1)	0.00009
>1 positive blood cultures	3 (14.3)	1 (4.2)	0.23
Polymicrobial bacteremia	0 (0)	10 (41.7)	0.0008
Positive cultures from other sites	8 (38.1)	1 (4.2)	0.005
Complications	7 (33.3)	1 (4.2)	0.01
Attributable mortality	4 (19.0)	0 (0)	0.025

^a^excluding pseudobacteremia.

^b^The percentages add up to more than 100% because some patients have more than one diagnosis.

^c^by Chi-square test.

**Table 2:** Clinical characteristics of patients with *Elizabethkingia* bacteremia.

Case/strain no.	Sex	Age	Underlying diseases	Diagnosis	Community- or hospital-acquired	No. of positive blood cultures (concomitant isolates)	Other culture-positive specimens	Antibiotic treatment	Complications/outcomee
*E. anophelis*									
EA1	M	54	DM, hyperlipidemia, ischemic cardiomyopathy	Nosocomial pneumonia	HA	1	None	Piperacillin-tazobactam	Acute pulmonary edema, CHF, survived
EA2	M	65	HT, DM, CAD, PVD, RAS, hyperlipidemia, carotid stenosis	Pseudobacteremia	NA	1	None	Cloxaciillin and levofloxacin, then amoxicillin-clavulanate	Survived
EA3	F	1m	Prematurity, twin, RDS, PDA	Catheter-related bacteremia	HA	3	None	Vancomycin, cefoperazone-sulbactam	Multi-organ failure, died
EA4	F	8d	Imperforated anus, rectovaginal fistula	Neonatal meningitis	HA	2	CSF	Vancomycin and rifampicin	Survived
EA5	M	65	COPD, CAD, CHF, AF, CRHD, right hip AVN	Community-acquired pneumonia	CA	1	Sputum	Ciprofloxacin	Survived
EA6	M	37	Dilated cardiomyopathy, CAD	Nosocomial bacteremia	HA	1	None	Ciprofloxacin	DIC, survived
EA7	F	64	HT, hyperlipidemia, stage 4 DLBC lymphoma	Neutropenic fever, severe mucositis	HA	2	None	Meropenem	Septic shock, haematemesis, died
EA8	M	68	HT, CRF, AAA	Nosocomial bacteremia	HA	1	Tracheal aspirate	Levofloxacin and vancomycin	Acute renal failure, died
EA9	M	73	CA hypopharynx, CA ampulla of Vater	Pseudobacteremia	NA	1	None	Cefuroxime	Survived
EA10	M	51	None	Community-acquired pneumonia	CA	1	Sputum	Ampicillin-sulbactam, then ciprofloxacin	Survived
EA11	F	104	HT, CAD, CVA, nephrotic syndrome	Community-acquired pneumonia	CA	1	None	Amoxicillin-clavulanate	Died
EA12	M	59	obesity, DM, CAD	Catheter-related bacteremia, drip site cellulitis	HA	1	None	Levofloxacin	Survived
EA13	F	35	Epilepsy, HT, ESRF on HD	Catheter-related bacteremia	HA	1	None	Levofloxacin	Survived
EA14	M	58	CRHD with AVR, ESRF on HD	Catheter-related bacteremia	HA	1	Catheter tip	Piperacillin-tazobactam, then levofloxacin	Septic shock, survived
EA15	M	88	COPD, old PTB	Nosocomial (aspiration) pneumonia	HA	1	None	Levofloxacin	Survived
HKU36	M	21d	None	Neonatal meningitis	HA	1	CSF	Vancomycin, piperacillin and rifampicin	Survived
HKU38	F	1d	Apnea of prematurity	Neonatal meningitis	HA	1	CSF	Vancomycin, piperacillin-tazobactam and rifampicin	metabolic acidosis, IVH, survived
*E. meningoseptica*									
EME1	M	51	Asthma, HT, scoliosis, MSSA infective spondylitis	Nosocomial bacteremia	HA	1	None	Ticarcillin-clavulanate	Survived
EME2	M	52	Biliary pancreatitis, RPC, cholecystectomy, cirrhosis with biliary stent	Post-cholangiogram acute cholangitis	HA	1	None	Levofloxacin and metronidazole	Survived
EME3	F	89	HT, AF, painless obstructive jaundice on palliative stenting	Biliary sepsis	CA	1	None	Levofloxacin	Survived
*E. miricola*									
EMI1	M	54	DM, hyperlipidemia, ischemic cardiomyopathy	Catheter-related bacteremia	HA	1	CVP catheter	Levofloxacin	Survived

^a^AAA, abdominal aortic aneurysm; AF, atrial fibrillation; AVN, avascular necrosis; AVR, atrial valve replacement; CA, carcinoma; CA, community-acquired; CAD, coronary artery disease; CHF, congestive heart failure; COPD; chronic obstructive pulmonary disease; CRF, chronic renal failure; CRHD, chronic rheumatic heart disease; CSF, cerebrospinal fluid; CVA, cerebrovascular accident; CVP, central venous pressure; DIC, disseminated intravascular coagulation; DLBC, diffuse large B cell; DM, diabetes mellitus; ESRF, end-stage renal failure; HA, hospital-acquired; HD, hemodialysis; HT, hypertension; IVH, intraventricular hemorrhage; MSSA, methicillin-sensitive *Staphylococcus aureus*; NA, not applicable; PDA, patent ductus arteriosus; PTB, pulmonary tuberculosis; PVD, peripheral vascular disease; RAS, renal artery stenosis; RDS, respiratory distress syndrome; RPC, recurrent pyogenic cholangitis.

**Table 3:** Clinical characteristics of patients with non-*Elizabethkingia* bacteremia.

Case/ strain no.	Sex	Age	Underlying diseases	Diagnosis	Community- or hospital-acquired	No. of positive blood cultures (concomitant isolates)	Other culture-positive specimens	Treatment + removal of catheter	Complications + outcome
Potentially novel *Chryseobacterium* species									
C13	M	64	Metastatic pancreatic carcinoma, sepsis	Postmortem pseudobacteremia	NA	1	None	NA	Died (non-attributable)
C15	M	68	Head injury	Pseudobacteremia	NA	1 (*Acinetobacter sp., Enterococcus faecium*)	No	Amoxicillin-clavulanate	Survived
*Chryseobacterium arthrosphaerae*									
C1	M	82	HT, gout, BPH, bilateral hydronephrosis and hydroureter	Nosocomial bacteremia	HA	1	None	Piperacillin-tazobactam, then levofloxacin	Survived
C2	F	48	Stage IV DLBC lymphoma	Catheter-related bacteremia, neutropenic fever	HA	6 (*Klebsiella pneumoniae, Acinetobacter baumanii*)	Central catheter	Imipenem-cilastatin and amikacin	Septic shock, survived
C9	M	56	COPD, pneumothorax	Nosocomial bacteremia	HA	1	None	Ticarcillin-clavulanate, levofloxacin	Survived
*Chryseobacterium gallinarum*									
C14	M	1	Cow milk allergy	Nosocomial bacteremia	HA	1	None	Piperacillin-tazobactam	Survived
*Chryseobacterium hominis*									
C8	F	76	DM, HT, CVA, CHF, AAA, PVD, hyperlipidemia	Pseudobacteremia	NA	1	None	None	Survived
C12	F	51	AML	Neutropenic fever	HA	1 (*Moraxella sp.*)	None	Piperacillin-tazobactam, vancomycin	Survived
*Chryseobacterium indologenes*									
C3	F	58	Hyperlipidemia, IgA nephropathy, Multiple myeloma	Pseudobacteremia	NA	1 (*Stenotrophomonas maltophila, Commamonas sp,, Ralstonia sp.*)	None	Levofloxacin, piperacillin-tazobactam	Survived
C4	F	49	DM, CRF, bilateral hydronephrosis, perinephric and psoas abscess	Pseudobacteremia	NA	1 (*Enterococcus facaelis, A. baumanii, Streptococcus mitis*)	None	Cefuroxime, ampicillin, levofloxacin	Survived
C5	M	78	HT, gout, CA caecum with metastases, thyroidectomy	Pseudobacteremia	NA	1 (*A. baumanii*)	None	Amoxicillin-clavulanate, ciprofloxacin	Survived
C6	F	57	CA rectum, mania, small bowel obstruction	Pseudobacteremia	NA	1 (*E. facaelis*, *A. baumanii*)	None	Amoxicillin-clavulanate	Survived
C7	M	84	HT, AF,CRHD, CVA, gout, BPH COPD	Pseudobacteremia	NA	1	None	Amoxicillin-clavulanate	Survived
C10	F	56	Acute encephalopathy, aspiration pneumonia, sepsis	Postmortem pseudobacteremia	NA	1 (*Pseuodmonas putida*, *Bacillus* sp., CNS, MSSA, non-hemolytic *Streptococcus*)	None	NA	Died (non-attributible)
*Chryseobacterium taihuense*									
C11	M	85	DM, DU, BPH	Pseudobacteremia	NA	1	None	Amoxicillin-clavulanate	Survived
*Flavobacterium lindanitolerans*									
F1	F	30	SLE, lupus nephritis	Nosocomial (post-transfusion) bacteremia	HA	1(*Bacillus sp.*)	None	Levofloxacin	Survived
*Planobacterium taklimakanense*									
P1	M	90	CAD, COPD, DM, BPH	Primary bacteremia	CA	1	None	Ceftriaxone	Survived
P2	M	1d	RDS, congenital pneumonia	Pseudobacteremia	NA	1	None	Penicillin and netilmicin	Survived
P3	F	99	COPD, CVA, intertrochanteric fracture	Pseudobacteremia	NA	1	None	Amoxicillin-clavulanate	Survived
P4	M	56	COPD, BPH, PTB	Pseudobacteremia	NA	1	None	Levofloxacin	Survived
P5	M	52	NPC, thyroidectomy, hypothyroidism	Nosocomial (post-transfusion) bacteremia	HA	1	None	Piperacillin-tazobactam	Survived
P6	F	1m	Sepsis, hypoglycemia	Pseudobacteremia	NA	1	None	Cefotaxime	Survived
*Sphingobacterium daejeonense*									
S1	M	94	COPD, cor pulmonale, BPH, gout, Shy-Drager syndrome, CAD	Pseudobacteremia	NA	1 (*K. pneumoniae*)	None	Cotrimoxazole	Survived
Potentially novel *Weeksella*-related species									
W1	F	18d	RSV pneumonia	Pseudobacteremia	NA	1	None	Ampicillin, netilmicin and erythromycin	Survived

^a^AAA, abdominal aortic aneurysm; AF, atrial fibrillation; AML, acute myeloid leukemia; BPH, benign prostatic hyperplasia; CA, carcinoma; CA, community-acquired; CAD, coronary artery disease; CHF, congestive heart failure; CNS, coagulase-negative *Staphylococcus*; COPD; chronic obstructive pulmonary disease; CRF, chronic renal failure; CRHD, chronic rheumatic heart disease; CVA, cerebrovascular accident; DLBC, diffuse large B cell; DM, diabetes mellitus; DU, Duodenal ulcer; HA, hospital-acquired; HT, hypertension; MSSA, methicillin-sensitive *Staphylococcus aureus*; NA, not applicable; NPC, nasopharyngeal carcinoma; PTB, pulmonary tuberculosis; PVD, peripheral vascular disease; RDS, respiratory distress syndrome; RSV, respiratory syncytial virus; SLE, systemic lupus erythematosus.

## References

[b1] Vandamme P., Bernardet J.-F., Kersters S. K. & Holmes B. New perspectives in the classification of the Flavobacteria: description of *Chryseobacterium* gen. nov., *Bergeyella* gen. nov., and *Empedobacter* nom. rev. Int. J. Syst. Evol. Microbiol. 44, 827–831 (1994).

[b2] Kim K. K., Kim M. K., Lim J. H., Park H. Y. & Lee S. T. Transfer of Chryseobacterium meningosepticum and Chryseobacterium miricola to *Elizabethkingia gen. nov.* as *Elizabethkingia meningoseptica comb. nov.* and *Elizabethkingia miricola comb. nov*. Int. J. Syst. Evol. Microbiol. 55, 1287–1293 (2005). 15879269 10.1099/ijs.0.63541-0

[b3] Kämpfer P., Busse H. J., McInroy J. A. & Glaeser S. P. *Elizabethkingia endophytica* sp. nov., isolated from Zea mays and emended description of *Elizabethkingia anophelis* Kämpfer *et al.* 2011. Int. J. Syst. Evol. Microbiol. 65, 2187–2193 (2015). 25858248 10.1099/ijs.0.000236

[b4] Bloch K. C., Nadarajah R. & Jacobs R. *Chryseobacterium meningosepticum*: an emerging pathogen among immunocompromised adults. Report of 6 cases and literature review. Medicine (Baltimore) 76, 30–41 (1997). 9064486 10.1097/00005792-199701000-00003

[b5] Weaver K. N. *et al.* Acute emergence of *Elizabethkingia meningoseptica* infection among mechanically ventilated patients in a long-term acute care facility. Infect. Control Hosp. Epidemiol. 31, 54–58 (2010). 19929372 10.1086/649223

[b6] Matyi S. A. *et al.* Draft genome sequences of *Elizabethkingia meningoseptica*. Genome Announc. 1, e00444–13 (2013). 23846266 10.1128/genomeA.00444-13 PMC3709143

[b7] Li Y. *et al.* *Chryseobacterium miricola* sp. nov., a novel species isolated from condensation water of space station Mir. Syst. Appl. Microbiol. 26, 523–528 (2003). 14666980 10.1078/072320203770865828

[b8] Green O., Murray P. & Gea-Banacloche J. C. Sepsis caused by *Elizabethkingia miricola* successfully treated with tigecycline and levofloxacin. Diagn. Microbiol. Infect. Dis. 62, 430–432 (2008). 18842380 10.1016/j.diagmicrobio.2008.07.015 PMC2650818

[b9] Kämpfer P. *et al.* *Elizabethkingia anophelis* sp. nov., isolated from the midgut of the mosquito *Anopheles gambiae*. Int. J. Syst. Evol. Microbiol. 61, 2670–2675 (2011). 21169462 10.1099/ijs.0.026393-0

[b10] Frank T. *et al.* First case of *Elizabethkingia anophelis* meningitis in the Central African Republic. Lancet 381, 1876 (2013). 23706804 10.1016/S0140-6736(13)60318-9

[b11] Teo J. *et al.* First case of *E anophelis* outbreak in an intensive-care unit. Lancet 382, 855–856 (2013). 24012265 10.1016/S0140-6736(13)61858-9

[b12] Lau S. K. *et al.* Evidence for *Elizabethkingia anophelis* transmission from mother to infant, Hong Kong. Emerg. Infect. Dis. 21, 232–241 (2015). 25625669 10.3201/eid2102.140623 PMC4313635

[b13] Bobossi-Serengbe G., Gody J. C., Beyam N. E. & Bercion R. First documented case of *Chryseobacterium meningosepticum* meningitis in Central African Republic. Med. Trop. (Mars) 66, 182–184 (2006). 16775944

[b14] Balm M. N. *et al.* Bad design, bad practices, bad bugs: frustrations in controlling an outbreak of *Elizabethkingia meningoseptica* in intensive care units. J. Hosp. Infect. 85, 134–140 (2013). 23958153 10.1016/j.jhin.2013.05.012

[b15] Kajla M. K., Andreeva O., Gilbreath T. M. 3rd & Paskewitz S. M. Characterization of expression, activity and role in antibacterial immunity of *Anopheles gambiae* lysozyme c-1. Comp. Biochem. Physiol. B Biochem. Mol. Biol. 155, 201–209 (2010). 19932188 10.1016/j.cbpb.2009.11.012 PMC2795013

[b16] Quick J., Constantinidou C., Pallen M. J., Oppenheim B. & Loman N. J. Draft genome sequence of *Elizabethkingia meningoseptica* isolated from a traumatic wound. Genome Announc. 2, e00355–14 (2014). 24812216 10.1128/genomeA.00355-14 PMC4014684

[b17] Luk W. K. *et al.* Inpatient emergencies encountered by an infectious disease consultative service. Clin. Infect. Dis. 26, 695–701 (1998). 9524847 10.1086/514591

[b18] Weinstein M. P. *et al.* The clinical significance of positive blood cultures in the 1990s: a prospective comprehensive evaluation of the microbiology, epidemiology, and outcome of bacteremia and fungemia in adults. Clin. Infect. Dis. 24, 584–602 (1997). 9145732 10.1093/clind/24.4.584

[b19] Versalovic J. *et al.* Manual of Clinical Microbiology, 10th ed. (ASM Press, 2011).

[b20] Lau S. K. P. *et al.* *Eggerthella hongkongensis* sp. nov. and *Eggerthella sinensis* sp. nov., two novel *Eggerthella* species, account for half of the cases of *Eggerthella* bacteremia. Diagn. Microbiol. Infect. Dis. 49, 255–263 (2004). 15313530 10.1016/j.diagmicrobio.2004.04.012

[b21] Tamura K., Stecher G., Peterson D., Filipski A. & Kumar S. MEGA6: Molecular Evolutionary Genetics Analysis version 6.0. Mol. Biol. Evol. 30, 2725–2729 (2013). 24132122 10.1093/molbev/mst197 PMC3840312

[b22] Lau S. K. *et al.* Matrix-assisted laser desorption ionization-time of flight mass spectrometry for rapid identification of *Burkholderia pseudomallei*: importance of expanding databases with pathogens endemic to different localities. J. Clin. Microbiol. 50, 3142–3143 (2012). 22718946 10.1128/JCM.01349-12 PMC3421815

[b23] Lau S. K. P. *et al.* Matrix-assisted laser desorption ionization time-of-flight mass spectrometry for identification of bacteria that are difficult to identify in clinical laboratories. J. Clin. Pathol. 67, 361–366 (2014). 24143023 10.1136/jclinpath-2013-201818

[b24] Tsang C. C. *et al.* Subcutaneous phaeohyphomycotic nodule due to *Phialemoniopsis hongkongensis* sp. nov. J. Clin. Microbiol. 52, 3280–3289 (2014). 24966363 10.1128/JCM.01592-14 PMC4313151

[b25] Clinical and Laboratory Standards Institute. M02-A11: performance standards for antimicrobial disk susceptibility tests; approved standard, 11^th^ edn. Wayne, Pa. (2012).

[b26] Woo P. C. *et al.* & *L. hongkongensis* study group. Association of *Laribacter hongkongensis* in community-acquired gastroenteritis with travel and eating fish: a multicentre case-control study. Lancet 363, 1941–1947 (2004). 15194253 10.1016/S0140-6736(04)16407-6

