# Supplementary material for: Elizabethkingia anophelis bacteremia is associated with clinically significant infections and high mortality
Source: Sci Rep. 2016 May 17;6:26045. doi: 10.1038/srep26045 (PMC4868968; doi:10.1038/srep26045)
Supplement: Supplementary Tables [file srep26045-s1.doc]

# *Elizabethkingia anophelis* bacteremia is associated with clinically significant infections and high mortality

Susanna K. P. Lau^1,2,3,4^*, Wang-Ngai Chow^1^, Chuen-Hing Foo^1^, Shirly O. T. Curreem^1^, George Chi-Shing Lo^1^, Jade L. L. Teng^1^, Jonathan H. K. Chen^1^, Ricky H. Y. Ng^5^, Alan K. L. Wu^6^, Ingrid Y. Y. Cheung^7^, Sandy K. Y. Chau^5^, David C. Lung^8^, Rodney A. Lee^6^, Cindy W. S. Tse^7^, Kitty S. C. Fung^5^, Tak-Lun Que^8^, and Patrick C. Y. Woo^1,2,3,4^*

^1^Department of Microbiology, ^2^State Key Laboratory of Emerging Infectious Diseases, ^3^Research Centre of Infection and Immunology, ^4^Carol Yu Centre for Infection, The University of Hong Kong, Hong Kong, China; ^5^Department of Pathology, United Christian Hospital, Hong Kong, China; ^6^Department of Pathology, Pamela Youde Nethersole Eastern Hospital, Hong Kong, China; ^7^Department of Pathology, Kwong Wah Hospital, Hong Kong, China; ^8^Department of Clinical Pathology, Tuen Mun Hospital, Hong Kong, China.

*Corresponding authors. Department of Microbiology, The University of Hong Kong, Room 423, University Pathology Building, Queen Mary Hospital Compound, Pokfulam Road, Hong Kong, China. Phone: (852) 22554892. Fax: (852) 28551241. Email: [skplau@hku.hk](mailto:skplau@hku.hk) (SKP Lau); [pcywoo@hku.hk](mailto:pcywoo@hku.hk) (PCY Woo)

**Supplementary TABLE 1** Identification of 45 *Elizabethkingia*-like blood culture isolates by 16S rRNA gene sequencing

| Strain no. | Identification by 16S rRNA gene sequencing | Closest GenBank match  (accession no.) | % nucleotide identity to closest match |
| --- | --- | --- | --- |
| EA1 | *Elizabethkingia anophelis* | *Elizabethkingia anophelis* R26^T^ (EF426425) | 99.9 |
| EA2 | *Elizabethkingia anophelis* | *Elizabethkingia anophelis* R26^T^ (EF426425) | 99.5 |
| EA3 | *Elizabethkingia anophelis* | *Elizabethkingia anophelis* R26^T^ (EF426425) | 99.8 |
| EA4 | *Elizabethkingia anophelis* | *Elizabethkingia anophelis* R26^T^ (EF426425) | 99.7 |
| EA5 | *Elizabethkingia anophelis* | *Elizabethkingia anophelis* R26^T^ (EF426425) | 99.8 |
| EA6 | *Elizabethkingia anophelis* | *Elizabethkingia anophelis* R26^T^ (EF426425) | 99.7 |
| EA7 | *Elizabethkingia anophelis* | *Elizabethkingia anophelis* R26^T^ (EF426425) | 99.6 |
| EA8 | *Elizabethkingia anophelis* | *Elizabethkingia anophelis* R26^T^ (EF426425) | 99.2 |
| EA9 | *Elizabethkingia anophelis* | *Elizabethkingia anophelis* R26^T^ (EF426425) | 99.6 |
| EA10 | *Elizabethkingia anophelis* | *Elizabethkingia anophelis* R26^T^ (EF426425) | 99.6 |
| EA11 | *Elizabethkingia anophelis* | *Elizabethkingia anophelis* R26^T^ (EF426425) | 99.9 |
| EA12 | *Elizabethkingia anophelis* | *Elizabethkingia anophelis* R26^T^ (EF426425) | 99.5 |
| EA13 | *Elizabethkingia anophelis* | *Elizabethkingia anophelis* R26^T^ (EF426425) | 99.7 |
| EA14 | *Elizabethkingia anophelis* | *Elizabethkingia anophelis* R26^T^ (EF426425) | 99 |
| EA15 | *Elizabethkingia anophelis* | *Elizabethkingia anophelis* R26^T^ (EF426425) | 99.4 |
| HKU36 | *Elizabethkingia anopheles* | *Elizabethkingia anophelis* R26^T^ (EF426425) | 99 |
| HKU38 | *Elizabethkingia anophelis* | *Elizabethkingia anophelis* R26^T^ (EF426425) | 99.8 |
| EME1 | *Elizabethkingia meningoseptica* | *Elizabethkingia meningoseptica* ATCC 13253^T^ (NR042267) | 99.8 |
| EME2 | *Elizabethkingia meningoseptica* | *Elizabethkingia meningoseptica* ATCC 13253^T^ (NR042267) | 99.4 |
| EME3 | *Elizabethkingia meningoseptica* | *Elizabethkingia meningoseptica* ATCC 13253^T^ (NR042267) | 99.8 |
| EMI1 | *Elizabethkingia miricola* | *Elizabethkingia miricola* LMG 22470^T^ (AB071953) | 99.5 |
| C1 | *Chryseobacterium arthrosphaerae* | *Chryseobacterium arthrosphaerae* CC-VM-7^T^ (FN398101) | 99.8 |
| C2 | *Chryseobacterium arthrosphaerae* | *Chryseobacterium arthrosphaerae* CC-VM-7^T^ (FN398101) | 99.8 |
| C3 | *Chryseobacterium indologenes* | *Chryseobacterium indologenes* LMG 8337^T^ (LN232813) | 99.1 |
| C4 | *Chryseobacterium indologenes* | *Chryseobacterium indologenes* LMG 8337^T^ (LN232813) | 99.7 |
| C5 | *Chryseobacterium indologenes* | *Chryseobacterium indologenes* LMG 8337^T^ (LN232813) | 99.6 |
| C6 | *Chryseobacterium indologenes* | *Chryseobacterium indologenes* LMG 8337^T^ (LN232813) | 99.7 |
| C7 | *Chryseobacterium indologenes* | *Chryseobacterium indologenes* LMG 8337^T^ (LN232813) | 99.6 |
| C8 | *Chryseobacterium hominis* | *Chryseobacterium hominis* NF802^T^ (AM261868) | 99.5 |
| C9 | *Chryseobacterium arthrosphaerae* | *Chryseobacterium arthrosphaerae* CC-VM-7^T^ (FN398101) | 99.6 |
| C10 | *Chryseobacterium indologenes* | *Chryseobacterium indologenes* LMG 8337^T^ (LN232813) | 99.9 |
| C11 | *Chryseobacterium taihuense* | *Chryseobacterium taihuense* THMBM1^T^ (JQ283114) | 99.1 |
| C12 | *Chryseobacterium hominis* | *Chryseobacterium hominis* NF802^T^ (AM261868) | 99.6 |
| C13 | Novel *Chryseobacterium* species | *Chryseobacterium taeanense* PHA3-4^T^ (AY883416) | 98.4 |
| C14 | *Chryseobacterium gallinarum* | *Chryseobacterium gallinarum* 100^T^ (KC494697) | 99.7 |
| C15 | Novel *Chryseobacterium* species | *Chryseobacterium hominis* NF802^T^ (AM261868) | 97.4 |
| F1 | *Flavobacterium lindanitolerans* | *Flavobacterium lindanitolerans* IP10^T^ (EF424395) | 99.6 |
| P1 | *Planobacterium taklimakanense* | *Planobacterium taklimakanense* X-65^T^ (EU718058) | 99 |
| P2 | *Planobacterium taklimakanense* | *Planobacterium taklimakanense* X-65^T^ (EU718058) | 99.7 |
| P3 | *Planobacterium taklimakanense* | *Planobacterium taklimakanense* X-65^T^ (EU718058) | 99.4 |
| P4 | *Planobacterium taklimakanense* | *Planobacterium taklimakanense* X-65^T^ (EU718058) | 99.6 |
| P5 | *Planobacterium taklimakanense* | *Planobacterium taklimakanense* X-65^T^ (EU718058) | 99.7 |
| P6 | *Planobacterium taklimakanense* | *Planobacterium taklimakanense* X-65^T^ (EU718058) | 99.5 |
| S1 | *Sphingobacterium daejeonense* | *Sphingobacterium daejeonense* TR6-04^T^ (AB249372) | 99.3 |
| W1 | Novel *Weeksella*-related species | *Weeksella virosa* DSM 16922^T^ (CP002455) | 91.9 |

**Supplementary TABLE 2** Phenotypic characteristics and antibiotic susceptibilities of 21 *Elizabethkingia* isolates and type strains of *E. anopehlis*, *E. meningoseptica* and *E. miricola*

| Phenotypic tests/ antibiotic susceptibilities^a^ | *E. anophelis*  (n=17) | *E. meningoseptica*  (n=3) | *E. miricola*  (n=1) | *E. anophelis*  R26^T^ | *E. meningoseptica* ATCC 13253^T^ | *E. miricola*  LMG 22470^T^ |
| --- | --- | --- | --- | --- | --- | --- |
| Citrate utilization | 23.5 | 0 | + | + | − | + |
| Urea hydrolysis | 17.6 | 0 | − | − | − | + |
| Cellobiose fermentation | 29.4 | 0 | + | + | − | + |
| Melibiose fermentation | 5.9 | 66.7 | − | − | + | + |
| Growth on MacConkey agar | 70.6 | 66.7 | + | + | + | + |
| Identification by  Vitek 2 GNI | *E. meningoseptica* (91-99% confidence) | *E. meningoseptica* (97% confidence) | *E. meningoseptica* (97% confidence) | *E. meningoseptica* (97% confidence) | *E. meningoseptica* (99% confidence) | *E. meningoseptica* (99% confidence) |
| MALDI-TOF MS using Bruker database^b^ | *E. meningoseptica* (n=10, 2.073 to 2.403)  *Elizabethkingia* sp. (n=2, 1.952 to 1.971)  No identification  (n=5, 1.32 to 1.42) | *E. meningoseptica* (2.354 to 2.415) | *E. miricola*  (2.162) | *E. meningoseptica*  (1.854) | *E. meningoseptica* (2.476) | *E. miricola*  (2.07) |
| MALDI-TOF MS using expanded database^b^ | *E. anophelis*  (n=17, 2.321 to 2.634) | *E. meningoseptica* (2.376 to 2.501) | *E. miricola*  (2.278) | *E. anophelis*  (2.531) | *E. meningoseptica* (2.433) | *E. miricola*  (2.131) |
| Ciprofloxacin | 100 | 100 | S | S | S | S |
| Cefoperazone-sulbactam | 100 | 100 | S | S | S | S |
| Ceftazidime | 5.9 | 0 | R | R | R | R |
| Piperacillin | 41.1 | 66.7 | S | S | S | S |
| Imipenem | 0 | 0 | R | R | R | R |
| Amikacin | 0 | 0 | R | R | R | R |
| Gentamicin | 0 | 0 | R | R | R | R |
| Tobramycin | 0 | 0 | R | R | R | R |
| Rifampicin | 58.8 | 66.7 | S | S | S | S |
| Cotrimoxazole | 70.6 | 100 | R | S | S | S |
| Vancomycin (30 µg) | 100 | 100 | S | S | S | S |

^a^Percentages of positive reactions or susceptibilities to antibiotics; +, positive reaction; −, negative reaction

^b^Number of isolates with the respective identification and score range are indicated in brackets
